# Association of Epigenetic Differences Screened in a Few Cases of Monozygotic Twins Discordant for Attention-Deficit Hyperactivity Disorder With Brain Structures

**DOI:** 10.3389/fnins.2021.799761

**Published:** 2022-01-21

**Authors:** Takashi X. Fujisawa, Shota Nishitani, Kai Makita, Akiko Yao, Shinichiro Takiguchi, Shoko Hamamura, Koji Shimada, Hidehiko Okazawa, Hideo Matsuzaki, Akemi Tomoda

**Affiliations:** ^1^Research Center for Child Mental Development, University of Fukui, Fukui, Japan; ^2^Division of Developmental Higher Brain Functions, United Graduate School of Child Development, Osaka University, Kanazawa University, Hamamatsu University School of Medicine, Chiba University, and University of Fukui, Osaka, Japan; ^3^Department of Child and Adolescent Psychological Medicine, University of Fukui Hospital, Fukui, Japan; ^4^Biomedical Imaging Research Center, University of Fukui, Fukui, Japan

**Keywords:** attention-deficit hyperactivity disorder (ADHD), DNA methylation, monozygotic twins, voxel-based morphometry (VBM), SorCS2

## Abstract

The present study examined the relationship between DNA methylation differences and variations in brain structures involved in the development of attention-deficit hyperactivity disorder (ADHD). First, we used monozygotic (MZ) twins discordant (2 pairs of 4 individuals, 2 boys, mean age 12.5 years) for ADHD to identify candidate DNA methylation sites involved in the development of ADHD. Next, we tried to replicate these candidates in a case-control study (ADHD: *N* = 18, 15 boys, mean age 10.0 years; Controls: *N* = 62, 40 boys, mean age 13.9 years). Finally, we examined how methylation rates at those sites relate to the degree of local structural alterations where significant differences were observed between cases and controls. As a result, we identified 61 candidate DNA methylation sites involved in ADHD development in two pairs of discordant MZ twins, among which elevated methylation at a site in the sortilin-related Vps10p domain containing receptor 2 (*SorCS2*) gene was replicated in the case-control study. We also observed that the ADHD group had significantly reduced gray matter volume (GMV) in the precentral and posterior orbital gyri compared to the control group and that this volume reduction was positively associated with *SorCS2* methylation. Furthermore, the reduced GMV regions in children with ADHD are involved in language processing and emotional control, while *SorCS2* methylation is also negatively associated with emotional behavioral problems in children. These results indicate that *SorCS2* methylation might mediate a reduced GMV in the precentral and posterior orbital gyri and therefore influence the pathology of children with ADHD.

## Introduction

Attention-deficit hyperactivity disorder (ADHD) is one of the most common mental disorders in childhood, characterized by inattention, hyperactivity, and impulsivity, according to the 5th edition of the Diagnostic and Statistical Manual of Mental Disorders (DSM-5) (American Psychiatric Association, 2013) and often reaching into adulthood. The prevalence of ADHD in children worldwide was estimated to be 7.2% in a meta-analysis of 175 studies (Thomas et al., 2015). Patients with ADHD have difficulties in various cognitive domains, such as cognitive control, attention, timing, and working memory (Biederman et al., 1991; Rubia, 2018), as well as in other domains involved in emotional processing, such as motivation and timing processing, such as timing dissociation and delay-related impairments (Biederman et al., 1993; Sonuga-Barke et al., 2010; Rubia, 2018). According to a prospective follow-up study, approximately 50% of children with ADHD continue to have symptoms until adulthood, and if left untreated, they can be at higher risk of psychiatric problems such as depression, substance abuse, and social problems such as unemployment and criminal offenses (Biederman et al., 2006; Molina et al., 2009).

While the heritability of ADHD has been reported to be as high as 72–88% (Larsson et al., 2014), there has been obvious discordance in ADHD diagnosis between monozygotic (MZ) twin pairs, and often differences in severity within MZ concordant cases (Larsson et al., 2014), suggesting that epigenetic factors may be involved in the etiology. Epigenetic modifications regulate gene expression independently of changes in DNA sequence, primarily through DNA methylation and histone modifications (Henikoff and Matzke, 1997), and have been suggested to serve as a critical link between external environmental factors and long-lasting phenotypic changes (Malki et al., 2016). Epigenetic changes in the brain have been found to be involved in cognitive neurological processes, including psychiatric disorders, neurogenesis, and brain development. Candidate gene studies on ADHD’s DNA methylation profile based on peripheral samples such as blood or saliva have shown different methylation patterns of genes involved not only in dopaminergic, serotonergic, and neurotrophic systems including *SLC6A4*, *DRD4*, *COMT*, *BDNF*, and *NGFR*, but also neurotransmitter release or neurite outgrowth including *ERC2* and *CREB5* and associated with the symptoms and severity of ADHD (van Mil et al., 2014; Park et al., 2015; Xu et al., 2015; Dadds et al., 2016; Heinrich et al., 2017; Sengupta et al., 2017; Neumann et al., 2020).

Using disease discordant MZ twins for comparison in epidemiological epigenetic studies would be an ideal strategy, because the sex, age, perinatal environment, and other shared environmental factors that significantly influence the epigenome should be matched within MZ twins (Bell and Spector, 2011). Recent findings have revealed considerable epigenetic differences between MZ twins (Kaminsky et al., 2009), and such differences have been associated with phenotypic discordance between MZ twins, including psychiatric disorders (Kuratomi et al., 2008; Sugawara et al., 2011; Wong et al., 2014; Malki et al., 2016). Regarding ADHD, Chen et al. (2018) recently examined the relationship between brain structure and whole blood DNA methylation in 14 pairs of MZ-discordant cases, finding structural alterations in the striatum and cerebellum, as well as significant epigenetic differences in genes, such as γ-aminobutyric acid (GABA), dopamine and serotonin neurotransmitter systems, in these “discordant” brain structures (Chen et al., 2018). These findings support the role of DNA methylation in ADHD. However, given the high heterogeneity of ADHD, not only the study of DNA methylation associated significantly different brain regions in MZ discordant twins, but also examining the association between methylation array data analysis and structural alterations in the whole brain, including the cerebral cortex, can comprehensively elucidate the complex pathology of ADHD.

Here, we examined the relationship between DNA methylation differences on array data and variations in brain structures involved in the development of ADHD. Thus, our main hypothesis was that the DNA methylation sites nominated using identical MZ twins discordant for ADHD are ADHD-specific, and significantly associated with brain structures and symptoms observed in children with ADHD compared to typically developing children.

## Materials and Methods

### Participants

Two pairs of MZ twins discordant for ADHD were recruited from the Department of Child and Adolescent Psychological Medicine at the University of Fukui Hospital. The twins were 9-year-old males (pair 1) and 16-year-old females (pair 2). Eighteen children with ADHD (16 males and 2 females, mean age = 9.7 ± 1.6 years) were also recruited at the department for the case-control study. The diagnosis of ADHD was assessed by licensed child and adolescent psychiatrists according to DSM-5 criteria (American Psychiatric Association, 2013). Participants were also administered an assessment module of DSM-IV ADHD from the Schedule of Affective Disorders and Schizophrenia for School-Age Children, Epidemiologic version (K-SADS-E; Orvaschel and Puig-Antich, 1994). To further assess the core symptoms of ADHD (e.g., inattentive and hyperactive/impulsive symptoms), for the pairs of MZ twins discordant for ADHD parents were asked to complete the ADHD Rating Scale (ADHD-RS) (DuPaul et al., 1998) for all children with ADHD in the case-control study, the Swanson, Nolan, and Pelham Rating Scale (SNAP-IV) (Swanson, 1992). To exclude other psychiatric conditions (e.g., anxiety disorder), subjects were administered the Mini-International Neuropsychiatric Interview for Children and Adolescents (MINI-KID; Sheehan et al., 2010) by two licensed pediatric-psychological clinicians. Two existing Cohorts of 62 children (Cohort 1: *n* = 28, 21 males and 7 females, mean age = 14.9 ± 1.8 years; Cohort 2: *n* = 34, 19 males and 15 females, mean age = 13.1 ± 2.9 years) recruited from the local community in our previous study were used as controls for the case-control study (Takiguchi et al., 2015; Shimada et al., submitted^1^). All children had normal or corrected vision and normal hearing. All children, with the exception of the non-ADHD twins, were assessed using the Wechsler Intelligence Scale for Children-Fourth Edition (WISC-IV; Wechsler, 2003) or the Wechsler Adult Intelligence Scale-Third Edition (WAIS-III; Wechsler, 1997) and excluded if they had a full-scale intelligence quotient (FSIQ) <70. They were also excluded if they had any history of substance abuse, recent substance use, head trauma with loss of consciousness, significant fetal exposure to alcohol or drugs, perinatal or neonatal complications, neurological disorders, or medical conditions that might adversely affect growth and development. In the case-control study, behavioral and emotional problems were assessed using the Child Behavior Checklist (CBCL) in all children with ADHD and controls in Cohort 1 (Achenbach and Rescorla, 2001), and with the Strength and Difficulties Questionnaire (SDQ) in controls in Cohort 2 (Goodman, 1997).

### Saliva Collection and DNA Extraction

Saliva samples were collected using the Oragene Discover OGR-500 kit (DNA Genotek Inc., Ottawa, ON, Canada). DNA was extracted using prepIT^®^•L2P reagent (DNA Genotek Inc.) and quantified using the Qubit™ dsDNA HS Assay Kit (Thermo Fisher Scientific Inc., Pittsburgh, PA, United States).

### DNA Methylation Array

Genomic DNA (500 ng) was bisulfite-treated for cytosine-to-thymine conversion using the EZ DNA Methylation-Gold kit (Zymo Research, Irvine, CA, United States). The DNA was then whole-genome amplified, fragmented, and hybridized to the Human MethylationEPIC BeadChip (Illumina Inc., San Diego, CA, United States). BeadChips were scanned using iSCAN (Illumina Inc.), and the methylation level (β value) was calculated for each queried CpG locus using the GenomeStudio Methylation Module software, followed by the Psychiatric Genomics Consortium-Epigenome-Wide Association Studies quality control pipeline (Ratanatharathorn et al., 2017). Using CpGassoc (Barfield et al., 2012), samples with probe detection call rates <90% and those with an average intensity value of either <50% of the experiment-wide sample mean or <2,000 arbitrary units were excluded. Probes with detection *P* > 0.001 or those based on <3 beads were set to missing as were probes cross-hybridizing between autosomes and sex chromosomes (Teschendorff et al., 2013). CpG sites with missing data for >10% of samples within the dataset were excluded from the analysis. Probes containing single nucleotide polymorphisms (based on 1000 Genomes) within 10 base pairs of the target CpG were maintained in each dataset but flagged and tracked throughout the analysis pipeline. This decision was based on the growing recognition that sequence variants can influence DNA methylation patterns throughout the genome (Smith et al., 2014). Normalization of probe distribution and background differences between Type I and Type II probes was conducted using beta mixture quantile normalization (Teschendorff et al., 2013) after background correction. We did not remove the batch effect at this stage either for (1) MZ twins discordant for ADHD (Proband: *N* = 2, Non-proband: *N* = 2) and (2) ADHD cases (*N* = 18)–controls (*N* = 62) study since (1) those samples were scanned within the same chip and the row positional balance was identical [Pair 1: row 5 (Proband) vs. row 6 (Non-proband), pair 2: row 7 (Non-proband) vs. row 8 (Proband)], and (2) batches were completely confounded with case-control group status [Case chip ID (6 batches): 205111140162, 205111140170, 205111140171, 205134980172, 205134980191, and 205134980192; Control chip ID (8 batches): 203748260078, 203748260085, 203755070101, 203755080004, 203757350003, 203757350018, 203757350022, and 203757350023]. In such a case, it is not possible to remove technical signals when batches are confounded with variables of interest, even by employing tools such as ComBat (Johnson et al., 2007). As suggested by Nygaard et al. (2016) and Price and Robinson (2018), we decided to use chips and rows as additional covariates in our linear model instead of adjusting for batch effects in the initial processing to avoid *P*-value inflation. After quality control, 807,253 probes and 794,661 probes remained for (1) MZ twins discordant for ADHD and (2) the ADHD case-control study, respectively. We confirmed whether pair 1 and pair 2 were MZ twins using 59 “rs” probes on the EPIC chip using the R package *ewastool* (Heiss and Just, 2018), and found an identical genetic background (agreement: 0.9999891 and 0.9999893, respectively). As saliva contains a heterogeneous mixture of cell types of differing proportions in each sample, we used the EpiDISH method (Teschendorff et al., 2017) to estimate the proportion of epithelial cells derived from salivary DNA and entered it as a covariate in our statistical models.

### Brain-Image Acquisition and Pre-processing

Image acquisition in the 52 participants in the case-control study (18 with ADHD, 34 controls in Cohort 2) was performed using a GE Signa PET/MR 3-Tesla scanner with an 8-channel head coil (GE Healthcare, Milwaukee, WI, United States). A T1-weighted anatomical dataset was obtained using a fast spoiled-gradient recalled imaging sequence (voxel size 1 × 1 × 1 mm, TE = 3.24 ms, TR = 8.46 ms, flip angle = 11°). Image acquisition for the other 28 controls in Cohort 1 in the case-control study was performed using a GE Discovery MR 750 3-Tesla scanner with a 32-channel head coil (GE Healthcare, Milwaukee, WI, United States). A T1-weighted anatomical dataset was obtained from each subject by a fast-spoiled gradient recalled imaging sequence (voxel size 1 × 1 × 1 mm, TE = 1.99 ms, TR = 6.38 ms, flip angle = 11°). VBM was performed as a global analytic approach using the Statistical Parametric Mapping version 12 software^2^ (Wellcome Department of Imaging Neuroscience, University College London, London, United Kingdom) implemented in MATLAB 2020b (Math Works Inc., Natick, MA, United States). T1-weighted images were segmented coarsely into gray matter (GM), white matter, cerebrospinal fluid, and skull/scalp compartments using tissue probability maps. The Diffeomorphic Anatomical Registration through Exponentiated Lie Algebra algorithm was applied to the segmented brain tissues to generate a study-specific template and to achieve an accurate inter-subject registration with improved realignment of smaller inner structures (Ashburner, 2007). The segmented GM images were spatially normalized, and written out with an isotropic voxel resolution of 1.5 mm. Any volume change induced by normalization was adjusted via a modulation algorithm. Spatially normalized GM images were smoothed by a Gaussian kernel of 6.2 mm full width at half maximum.

### Statistical Analyses

First, to clarify epigenetic associations between proband and non-proband ADHD discordant MZ twins from methylation array data, multiple regression analysis was performed using CpGassoc (Barfield et al., 2012). In this analysis, DNA methylation at each CpG probe was entered as a dependent variable, and each group (proband or non-proband) entered as an independent variable. The proportion of epithelial cells was entered as a covariate, but we did not use age and sex as covariates because they were identical between groups.

Second, to confirm the reproducibility of the probes from the MZ twin discordant pair analysis in the case-control analysis, we examined the subset probes threshold set at *P* < 5.0E-05. DNA methylation at each CpG probe was entered as a dependent variable, and each group (case or control) as an independent variable. Age, sex, FSIQ, the proportion of epithelial cells, chip, and row for batch effect adjustments as explained previously, were entered as covariates, and results were threshold at false discovery rate (FDR) <0.05 by Benjamini-Hochberg.

Third, regional differences in gray matter volume (GMV) between groups were analyzed in SPM 12 using two-sample *t*-test models. Potential confounding effects of age, sex, FSIQ, scanner, and total GMV were modeled, and their attributed variances excluded from further analysis. Total GMV was calculated from the GM images obtained from pre-processing segmentation using the “Tissue Volumes” utility from the batching system in SPM12. A GM majority optimal threshold mask, created based on a study-specific sample, was applied to the analyses to eliminate voxels of non-GM for GMV-analyses (Ridgway et al., 2009). The resulting set of voxel values used for comparison generated a statistical parametric map of the t-statistic SPM{t} that was transformed to a unit normal distribution (SPM{Z}). The statistical threshold was set at *P* < 0.001 at the voxel level and *P* < 0.05, with a family wise error (FWE) correction for multiple comparisons. The anatomical localization of significant clusters was investigated using automated anatomical labeling and Brodmann area atlases implemented in the MRIcron software package (Rorden et al., 2007).

Finally, to further examine whether the ADHD-related GMV alterations were associated with DNA methylation, a correlation analysis for the residuals of each β value (methylation, GMV) controlled by control variables was performed. To this end, the adjusted eigenvariates, representing linearly transformed estimates of GMV, were extracted from the identified cluster. The significance level was set at *P* < 0.05. All statistical analyses were performed with R 3.6.3 (R Core Team, 2020), SPM 12, IBM SPSS Statistics for Windows version 26.0. (Armonk, NY: IBM Corp.).

### Meta-Analytic Decoding of Regional Function Using NeuroSynth

The functional properties of structural regions with alterations between groups were decoded using a large-scale database-informed meta-analytic approach as implemented in NeuroSynth (Yarkoni et al., 2011). A meta-analytic map associated with the identified region coordinates was derived. Further, the terms (excluding terms for brain regions) ranked by the z-score were visualized using an online word cloud generator^3^.

## Results

### Clinical Status in Monozygotic Twin Discordant Pairs

The clinical status of the MZ twin discordant pairs is shown in Table 1. First, regarding IQ, both pairs of children with ADHD were in the 25–75 percentile range, and no significant defects in cognitive ability were observed. Next, regarding ADHD symptoms, the inattentiveness score was >90th percentile in both ADHD children, and <75th percentile in both control children, suggesting that the inattention symptoms were significantly stronger in children with ADHD. The hyperactive/impulsive score was <50th percentile in both ADHD and control children, suggesting that there were no significant hyperactivity symptoms.

**TABLE 1:** Clinical status between monozygotic twin discordant pairs.

	Pair 1		Pair 2	
	ADHD	Control	ADHD	Control
Full Scale IQ (percentile)	109 (73)	–	97 (42)	–
ADHD Rating Scale-IV				
Inattentive score (percentile)	16 (94–95)	6 (50–75)	12 (92–93)	3 (50)
Hyperactive/impulsive score (percentile)	3 (25)	0 (1)	1 (25–50)	0 (1)

*IQ, intelligence quotient; ADHD, attention-deficit hyperactivity disorder.*

### Methylation Array Data Analysis of Monozygotic Twin Discordant Pairs

No CpG probes were detected above the genome-wide significance level under the number of EPIC array probes (*P* < 9.0E-08) (Tsai and Bell, 2015; Saffari et al., 2018) as a natural consequence of the extremely small sample size. We then threshold at *P* < 5.0E-05 (−log(*P*) = 4.3) by visual inspection of the Q-Q plot because the top probes over the threshold had residuals that steeply deviated from the expected line (Supplementary Figure 1). Sixty-one probes were above the threshold (Table 2 and Supplementary Figure 2), which were confirmed to be associated with ADHD in an independent case-control dataset.

**TABLE 2:** Top 61 differentially methylated CpG sites identified in ADHD-discordant monozygotic twin pairs, ranked by statistical significance and mean Δβ (calculated as DNA methylation level of control twin minus ADHD twin).

Rank	Probe ID	Gene	Chromosome	Position	Mean Δβ	*P*-value
1	cg19181132	*LRIG3*	12	59314527	0.0059	2.95E-07
2	cg00416255		15	58013677	−0.0021	7.39E-07
3	cg25395120	*KANSL1*	17	44111045	−0.0112	8.95E-07
4	cg04850211	*OBSCN*	1	228464232	0.0002	9.05E-07
5	cg02322229	*SYNE2;MIR548AZ*	14	64669716	−0.0097	1.74E-06
6	cg05803913	*MID1IP1*	X	38664442	0.0438	2.64E-06
7	cg24088250		17	75252179	0.0316	2.76E-06
8	cg03220187	*RFWD3*	16	74700945	0.0018	3.13E-06
9	cg26160626		7	155264300	−0.0055	4.68E-06
10	cg24972947	*ZNRF1*	16	75096111	−0.0248	5.83E-06
11	cg04430024	*MICAL1*	6	109778491	0.0769	6.04E-06
12	cg19670431	*SORCS2*	4	7436874	0.0454	6.77E-06
13	cg08676438	*ONECUT3*	19	1763695	0.0198	6.87E-06
14	cg23148094	*PNLIPRP1*	10	118350608	−0.0118	7.31E-06
15	cg26360087	*DCAKD*	17	43128925	0.0020	8.71E-06
16	cg06052716		7	1280607	0.0592	1.05E-05
17	cg10449882	*TMTC1*	12	29936815	−0.0043	1.08E-05
18	cg03700121	*CCDC86*	11	60610004	0.0192	1.13E-05
19	cg22641072	*CARD14*	17	78143724	0.0933	1.18E-05
20	cg09154309		2	170963987	−0.0068	1.21E-05
21	cg06111526	*ATF6B*	6	32086782	0.0062	1.33E-05
22	cg06385383	*CAND1.11*	11	10404199	0.0357	1.39E-05
23	cg05833251	*NGDN*	14	23938784	0.0048	1.54E-05
24	cg02860602		12	103356060	0.0017	1.63E-05
25	cg16524139	*TCF3*	19	1651573	0.0062	2.22E-05
26	cg03195600	*SOCS1*	16	11350371	0.0594	2.24E-05
27	cg13304638	*TBCD*	17	80834089	0.0013	2.26E-05
28	cg18007455	*LARGE*	22	34257627	0.0112	2.36E-05
29	cg03832293	*USP10*	16	84780698	−0.0124	2.48E-05
30	cg26436583	*PSTPIP2*	18	43649176	0.0148	2.70E-05
31	cg12326749	*SLC25A27*	6	46645430	−0.0066	2.94E-05
32	cg12109797		8	86414553	0.0099	3.07E-05
33	cg15451698		1	111257101	0.0179	3.25E-05
34	cg23098235	*HOXB3*	17	46634466	0.0265	3.27E-05
35	cg18430990	*TMEM240*	1	1475941	0.0068	3.28E-05
36	cg08833670	*ROBO3*	11	124746754	0.0197	3.40E-05
37	cg26486111		16	79646944	0.0009	3.40E-05
38	cg16475887	*MAPKBP1*	15	42102305	−0.0106	3.41E-05
39	cg08254353	*TMEM98*	17	31254670	0.0036	3.75E-05
40	cg17547033	*FMNL2*	2	153362087	−0.0272	3.76E-05
41	cg12507363	*DTNA*	18	32307046	−0.0346	3.78E-05
42	cg00411843		X	139584448	−0.0004	3.79E-05
43	cg25019889	*C1orf146*	1	92696685	−0.0113	3.82E-05
44	cg01980222	*TREM2*	6	41130917	0.0066	3.85E-05
45	cg02753619	*FBXW9*	19	12807497	0.0012	3.87E-05
46	cg07425090	*SYNCRIP*	6	86353447	0.0055	4.07E-05
47	cg09973105	*RNF175*	4	154681532	0.0252	4.27E-05
48	cg00966255		16	10479562	−0.0096	4.29E-05
49	cg13390059		1	88261783	−0.0277	4.40E-05
50	cg12437809	*FMO2*	1	171153544	−0.0038	4.56E-05
51	cg00025405		5	2135863	−0.0305	4.58E-05
52	cg18265326		16	65635738	0.0499	4.59E-05
53	cg20305576	*FLT1*	13	28968481	−0.0123	4.62E-05
54	cg08973053		8	120400084	−0.0073	4.65E-05
55	cg14028653	*CDH4*	20	60448776	−0.0081	4.67E-05
56	cg05600626	*CCDC116*	22	21991401	−0.0142	4.78E-05
57	cg25259707	*SNAPIN*	1	153630934	0.1102	4.82E-05
58	cg11476326		7	139245575	−0.0041	4.88E-05
59	cg19269246		2	42325942	0.0327	4.92E-05
60	cg19851976	*CRTC1*	19	18812192	0.0262	4.92E-05
61	cg12810354	*GABRA4*	4	46996987	−0.0028	4.93E-05

*ADHD, attention-deficit hyperactivity disorder.*

### Demographic and Questionnaire Data in the Case-Control Study

The ADHD and control groups were matched for sex and handedness, but there was a significant difference in age between groups (*T*(78) = 5.88, *P* < 0.001). A two-sample *t*-test was used to compare the total FSIQ and CBCL total scores between groups. Compared to the control group, the ADHD group showed lower FSIQ (*T*(78) = 3.05, *P* = 0.003) and higher levels of ADHD-related emotional and behavioral problems (CBCL total, *T*(44) = −8.90, *P* < 0.001), although data for CBCL was only available from Cohort 1. In addition, in the control group of Cohort 2, SDQ total scores were not significantly different from the mean of standard Japanese children using one-sample *t*-test (SDQ total, *T*(33) = 0.47, *P* = 0.641), suggesting no notable emotional and behavioral problems. Multiple regression analysis was also performed to examine the effect of age on the differences in FSIQ and CBCL total score between groups, with each variable as the dependent variable; the results showed that the effect of group was significant (FSIQ: β = −0.38, *T* = −2.96, *P* = 0.004; CBCL total: β = 0.93, *T* = 5.95, *P* < 0.001) while that of age was not (FSIQ: β = −0.10, *T* = −0.77, *P* = 0.445; CBCL total: β = 0.16, *T* = 0.99, *P* = 0.326). Regarding ADHD symptoms, the inattention score of SNAP-IV in the ADHD group showed clinically mild symptoms on average, and the hyperactivity/impulsivity and opposition/defiance scores did not reach clinically significant levels. Since the ratio of epithelial cells in saliva samples affects the estimated methylation level (β value), we analyzed for the difference between groups finding no significant difference between the ADHD and control groups. We also estimated GMV, WMV, and total brain volume in the ADHD and control groups, respectively, but found no differences between groups in any of these parameters (Table 3).

**TABLE 3:** Demographic and clinical characteristics of ADHD and control groups.

	ADHD (*n* = 18)	Controls (*n* = 62)	Statistics	*P-*value
Age (years), *Mean* (*SD*)	10.00 (1.61)	13.93 (2.69)	*t*(78) = 5.88	<0.001
Gender, *n* (male/female)	15/3	40/22	χ^2^(1) = 2.30	0.129
Handedness, *n* (right/left)	16/2	58/4	χ^2^(1) = 0.44	0.509
Full Scale IQ, *Mean* (*SD*)	98.11 (11.66)	106.98 (10.64)	*t*(78) = 3.05	0.003
CBCL Total^a^, *Mean* (*SD*)	69.06 (7.02)	47.93 (8.34)	*t*(44) = −8.90	<0.001
SDQ Total^b^, *Mean* (*SD*)	–	6.62 (4.28)		
SNAP-IV				
Inattention, *Mean* (*SD*)	16.16 (4.87)	–		
Hyperactivity/impulsivity, *Mean* (*SD*)	9.76 (5.21)	–		
Opposition/Defiance, *Mean* (*SD*)	7.76 (5.15)	–		
Epithelial Cells (%), *Mean* (*SD*)	28.47 (10.57)	28.63 (13.26)	*t*(78) = 0.40	0.961
Total GMV (ml), *Mean* (*SD*)	847.37 (48.94)	829.90 (74.70)	*t*(78) = 1.08	0.353
Total WMV (ml), *Mean* (*SD*)	378.93 (40.26)	392.04 (46.67)	*t*(78) = −0.15	0.283
Total Brain volume (ml), *Mean* (*SD*)	1226.30 (80.14)	1221.94 (133.16)	*t*(78) = 0.05	0.879

*ADHD, attention-deficit hyperactivity disorder; IQ, intelligence quotient; CBCL, Child Behavior Checklist; SDQ, Strength and Difficulties Questionnaire; SNAP-IV, Swanson, Nolan, and Pelham questionnaire GMV, gray matter volume; WMV, white matter volume.*

*^a^Data for the control group was obtained only for Cohort 1 (n = 28).*

*^b^Data obtained only from the control group of Cohort 2 (n = 34).*

### Case-Control Subset Methylation Analysis

We extracted the top 61 probes from the initial analysis, among which 60 probes meeting the quality control criteria were included in the case-control dataset. Among these 60 probes, three probes (cg03700121, cg18430990, and cg19670431) in the coiled-coil domain containing 86 (*CCDC86*), transmembrane protein 240 (*TMEM240*), and sortilin-related Vps10p domain containing receptor 2 (*SorCS2*) genes were significantly associated with ADHD (FDRs < 0.05, Table 4). However, two of these probes (cg03700121 and cg18430990) were inconsistent regarding the direction of the effect, with increased methylation in twin studies and decreased methylation in case-control studies, while only the probe involved in *SorCS2* showed consistent results in both studies. Hence, we used cg19670431 for subsequent epigenetic imaging analyses.

**TABLE 4:** Three CpG sites replicated in a sample of ADHD cases vs. controls.

Probe ID	Gene	Chromosome	Position	Mean Δβ	*P*-value (FDR)
cg03700121	*CCDC86*	11	60610004	−0.1208	0.0000015
cg18430990	*TMEM240*	1	1475941	−0.0301	0.0003126
cg19670431	*SORCS2*	4	7436874	0.0230	0.0171530

*FDR-adjusted P-values are corrected for multiple comparisons with 60 probes.*

*FDR, false discovery rate.*

### Structural Brain-Image Data in the Case-Control Study

**FIGURE 1:**
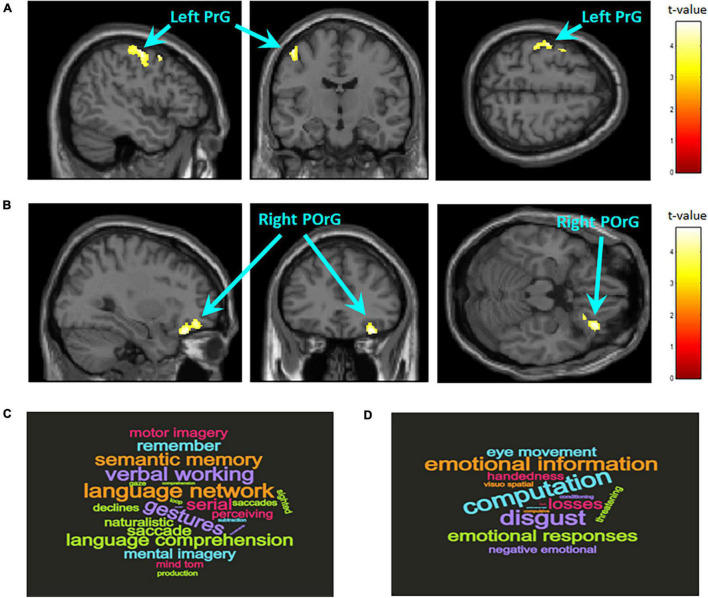
Brain regions with significantly larger gray matter volume in the control group compared to the ADHD group. The statistical threshold for the contrasts was set at voxel-level *P* < 0.001 uncorrected for height and cluster-level *P* < 0.05 family wise error rate corrected for multiple comparisons. The color bar denotes the *t*-statistic range. **(A)** Left precentral gyrus (PrG; BA6; MNI coordinates, *x* = –44, *y* = 5, *z* = 57; cluster size = 437 voxels). **(B)** Right posterior orbital gyrus (POrG; BA47; MNI coordinates, *x* = 24, *y* = 21, *z* = –21; cluster size = 555 voxels). Reverse inference of functional properties in the left PrG **(C)** and right POrG **(D)** as decoded by NeuroSynth. The font size represents the rank according to the strength of the relationship between regions and terms.

A whole-brain analysis with FWE correction at the cluster level was conducted to examine regional differences in GMV between the two groups (ADHD: *n* = 18, Controls: *n* = 62). Compared with the control group, the ADHD group showed reduced GMV in the left precentral gyrus (BA6; MNI coordinates, *x* = −44, *y* = 5, *z* = 57; cluster size = 437 voxels, *P* = 0.043, FWE corrected cluster level; Figure 1A) and the right posterior orbital gyrus (BA47; MNI coordinates, *x* = 24, *y* = 21, *z* = −21; cluster size = 555 voxels, *P* = 0.016, FWE corrected cluster level; Figure 1B).

Reverse inference on the functional properties related to the local regions where structural differences between groups were observed showed that most of the terms related to the left precentral gyrus are related to language functions such as semantic memory, working memory, theory of mind, and motion imagination (Figure 1C); most of the terms related to the right posterior orbital gyrus are related to–mainly negative–emotional information and their regulation (Figure 1D).

### Relationship Between Sortilin-Related Vps10p Domain Containing Receptor 2 Methylation and Brain Structural Alterations

**FIGURE 2:**
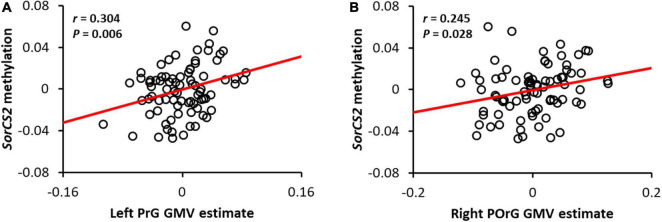
Correlation between *SorCS2* methylation and gray matter volume in the left precentral gyrus **(A)** and the right posterior orbital gyrus **(B)**. The red line shows the regression line. Adjusted residuals with respective covariates were used as values for methylation and local GMV of SorCS2. Note that the adjusted residuals for each covariate were used as estimates of *SorCS2* methylation and local GMV.

Sortilin-related Vps10p domain containing receptor 2 methylation was both positively correlated with GMV within a cluster in the precentral gyrus and the posterior orbital gyrus (Figures 2A,B). This result suggests that the more methylated *SorCS2* is, the larger the GMV of the precentral and posterior orbital gyri. In addition, to verify the tissue specificity of the methylation pattern, we examined the brain-saliva correlation for the CpG probe (cg19670431) identified using a web tool based on human samples (Braun et al., 2019) and also confirmed a trend toward a positive correlation (ρ = 0.37, *P* = 0.09).

### Relationship Between Clinical Symptom Scores, Sortilin-Related Vps10p Domain Containing Receptor 2 Methylation and Brain Structural Alterations

We performed a correlation analysis investigate the association between the neurobiological basis of both *SorCS2* methylation and local GMV alterations, and clinical symptoms (core symptoms based on SNAP-IV and emotional behavior problems based on CBCL tests) associated with ADHD. As a result, a significant negative correlation was confirmed between *SorCS2* methylation and CBCL total score (*r* = −0.444, *P* = 0.002), while no significant correlation was found with SNAP total score (*r* = −0.227, *P* = 0.366); these scores also showed no correlation with both local GMV alterations. These results suggest that *SorCS2* methylation may be involved in regulating emotional behavioral problems in children rather than ADHD-specific core symptoms.

## Discussion

This study investigated the relationship between DNA methylation differences based on array data and brain structure involved variations in the development of ADHD. First, we investigated two pairs of MZ twins discordant for ADHD and identified 61 candidates for DNA methylation sites involved in the development of ADHD. Next, using these candidates in a case-control study we found that children with ADHD had elevated methylation in the *SorCS2* gene body region. Finally, we observed that the ADHD group had significantly reduced GMV in the precentral gyrus and posterior orbital gyrus compared to the control group and that this volume reduction was positively associated with *SorCS2* methylation. In addition, the reduced GMV regions in children with ADHD are involved in language processing and emotional control, and *SorCS2* methylation is also negatively associated with emotional behavioral problems in children. These results indicate that *SorCS2* methylation might mediate a reduced GMV in the precentral and posterior orbital gyri and therefore influence the pathology of children with ADHD.

We suggested that SorCS2 methylation is involved in ADHD through methylation array analysis of MZ twin discordant cases and case-control groups, while previous epigenome-wide studies found no evidence that *SorCS2* methylation is involved in either children or adults with ADHD (van Dongen et al., 2019; Neumann et al., 2020; Rovira et al., 2020). Although SorCS2 is known to play a crucial role in neuronal viability and function (Glerup et al., 2016), human epidemiological studies have reported that single nucleotide polymorphisms in *SorCS2* are associated with the risk of developing psychiatric disorders such as ADHD (Alemany et al., 2015), bipolar disorder (Baum et al., 2008; Ollila et al., 2009), and schizophrenia (Christoforou et al., 2011). Although recent human genome-wide association studies have suggested that a gene set related to dopamine signaling is involved not only in ADHD alone but also in the comorbidity of ADHD with obesity and narcolepsy (Mota et al., 2020; Takahashi et al., 2020); in animal studies, lack of *SorCS2* reportedly induces ADHD-like behavior by altering the novelty response to psychostimulants and altering the dopaminergic firing pattern of the ventral tegmental area (Olsen et al., 2021). Other recent studies have revealed the significant roles of SorCS2 in brain derived neurotrophic factor (BDNF)-dependent plasticity and for social memory formation by *N*-methyl-D-aspartic (NMDA) receptor trafficking in hippocampal neurons (Glerup et al., 2016; Yang et al., 2021); in parallel, working memory deficits (Martinussen et al., 2005; Sowerby et al., 2011) and the involvement of BDNF or NMDA receptor signaling in ADHD have also been suggested (Bergman et al., 2011; Chang et al., 2014). Taken together, our results suggest that the *SorCS2* gene methylation found in this study may affect the development and certain symptoms of ADHD by affecting dopaminergic, BDNF, and/or NMDA receptor signaling pathways.

Sortilin-related Vps10p domain containing receptor 2 methylation was positively associated with GMV in the precentral and posterior orbital gyri in the ADHD group, suggesting that unmethylated *SorCS2* may lead to lower GMV. These results replicate prior results of surface area reduction in the left precentral and right orbital gyri found in another cohort of children with ADHD (Jung et al., 2019), as well as previous findings of cortical thickness reduction in the precentral gyrus and orbital gyri in a large-scale clinical sample of children (Hoogman et al., 2019). Big data analysis of structural magnetic resonance imaging of about 6,800 children found that the precentral gyrus surface area was one of the prominent local areas negatively associated with ADHD symptoms (Owens et al., 2021). Reverse inference showed that the precentral gyrus was associated with language function, and that children with ADHD have a higher risk of language problems (Sowerby et al., 2011; Hawkins et al., 2016; Korrel et al., 2017) which contributes to poor academic functioning (Sciberras et al., 2014). Hence, these findings suggest that *SorCS2* gene methylation may induce language-related difficulties in children with ADHD via reduced GMV in the precentral gyrus. Next, reductions in GMV and cortical thickness in the right orbital gyrus of individuals with ADHD have often been reported (Vaidya, 2012; Hoogman et al., 2019; Jung et al., 2019). Although the function of the orbital gyri appears related to emotional information and calculations, emotional information may play an important role in decision-making and executive function (Bechara et al., 2000; Rolls and Grabenhorst, 2008). Numerous imaging studies using emotional and executive function tasks have reported reduced functional activation in the right orbital gyrus (Epstein et al., 2007; Rubia et al., 2009; Cubillo et al., 2012; Godinez et al., 2015). Taken together, these findings suggest that *SorCS2* gene methylation may induce both reduced GMV in the orbital gyrus and emotional behavioral problems, although the direct association between the two could not be confirmed in this study.

Several limitations of the present study should be noted and taken into consideration for future studies. First, the sample size in this study was relatively small, including only two pairs of discordant MZ twins and 18 subjects in the ADHD group in the case-control study. In particular, the CpG sites identified in the array analysis of MZ twin discordant cases did not reach genome-wide significance level due to the small sample size; we only selected the top candidates based on their statistics. Although it is necessary to replicate the results with a larger sample size, it was particularly difficult to recruit MZ twins discordant by a single institution. Next, because we analyzed salivary DNA methylation, our data may not necessarily reflect the state of the brain due to the tissue specificity of methylation patterns (Smith et al., 2015). Regarding the tissue specificity of DNA methylation, although we tried to validate our results using a web tool that can investigate the correlation between methylation of brain, blood, saliva, and buccal cells collected from the same living human (Braun et al., 2019), another way to overcome the issue of tissue specificity is to directly examine the association between the methylation profile of *SorCS2* and the precentral or posterior orbital gyri in the postmortem brains of children with ADHD. Finally, in the case-control study, the children’s age and cognitive abilities did not match and the batches for array analysis and the MR scanners for brain imaging were confounding between groups because we used two existing cohorts as control group. Although these factors were used as control variables in the statistical analysis, it made it difficult to distinguish whether the association between *SorCS2* methylation and local GMV reduction was involved in the pathophysiology of ADHD or derived from demographic factors such as age and general cognitive abilities or research artifacts by batch and scanner effects. Although no significant difference was observed between the two groups in age-sensitive brain volume, there was a significant difference in emotional behavioral problems even after controlling for age, thus, future studies need to match age and cognitive abilities and exclude batch and scanner effects. Despite these limitations, this study sheds light on the some of the pathological mechanisms of ADHD in that it suggests DNA methylation candidates associated with brain structure specific to children with ADHD.

In conclusion, this study suggests that DNA methylation of the *SorCS2* gene may induce language-related and emotional behavioral problems via brain structure alterations specific to children with ADHD. Some pharmacological or psychosocial interventions that enhance *SorCS2* gene methylation may improve ADHD symptoms by interfering with the GMV reduction in the precentral and posterior orbital gyri. In the future, the elucidation of the molecular mechanism of local brain volume changes induced by *SorCS2* methylation will be useful for understanding the pathophysiology of ADHD.

## Data Availability Statement

The DNA methylation microarray data of monozygotic twins discordant samples have been deposited in the Gene Expression Omnibus database (GEO) with the primary accession code GSE186339 (https://www.ncbi.nlm.nih.gov/geo/query/acc.cgi? acc=GSE186339). Other data that support the results of this study are included in the article/Supplementary Material, further inquiries can be directed to the corresponding author.

## Ethics Statement

The study protocol was approved by the Research Ethics Committee of the University of Fukui, Japan, and all procedures were conducted in accordance with the Declaration of Helsinki and the Ethical Guidelines for Clinical Studies of the Ministry of Health, Labour, and Welfare of Japan. All parents provided written informed consent for participation in the study.

## Author Contributions

TF, SN, and AT contributed to the conception and design of the study, performed the experiments and statistical data analysis, and wrote the first draft of the manuscript. KM, AY, ST, SH, KS, HO, and HM contributed substantially to performing the experiments and collecting the data, revised the manuscript critically for intellectual content, and approved the submitted version. All authors contributed to the article and approved the submitted version.

## Conflict of Interest

The authors declare that the research was conducted in the absence of any commercial or financial relationships that could be construed as a potential conflict of interest.

## Publisher’s Note

All claims expressed in this article are solely those of the authors and do not necessarily represent those of their affiliated organizations, or those of the publisher, the editors and the reviewers. Any product that may be evaluated in this article, or claim that may be made by its manufacturer, is not guaranteed or endorsed by the publisher.

## Footnotes

1 Shimada, K., Hamamura, S., Takiguchi, S., Makita, K., Yao, A., Fujisawa, T. X., et al. (submitted). Developmental differences in resting-state functional connectivity among youth in small-group family-like residential care.

2
http://www.fil.ion.ucl.ac.uk/spm/software/spm12/

3
https://www.wordclouds.com
