# Supplementary figures and images for: Association of Epigenetic Differences Screened in a Few Cases of Monozygotic Twins Discordant for Attention-Deficit Hyperactivity Disorder With Brain Structures
Source: Front Neurosci. 2022 Jan 21;15:799761. doi: 10.3389/fnins.2021.799761 (PMC8823258; doi:10.3389/fnins.2021.799761)

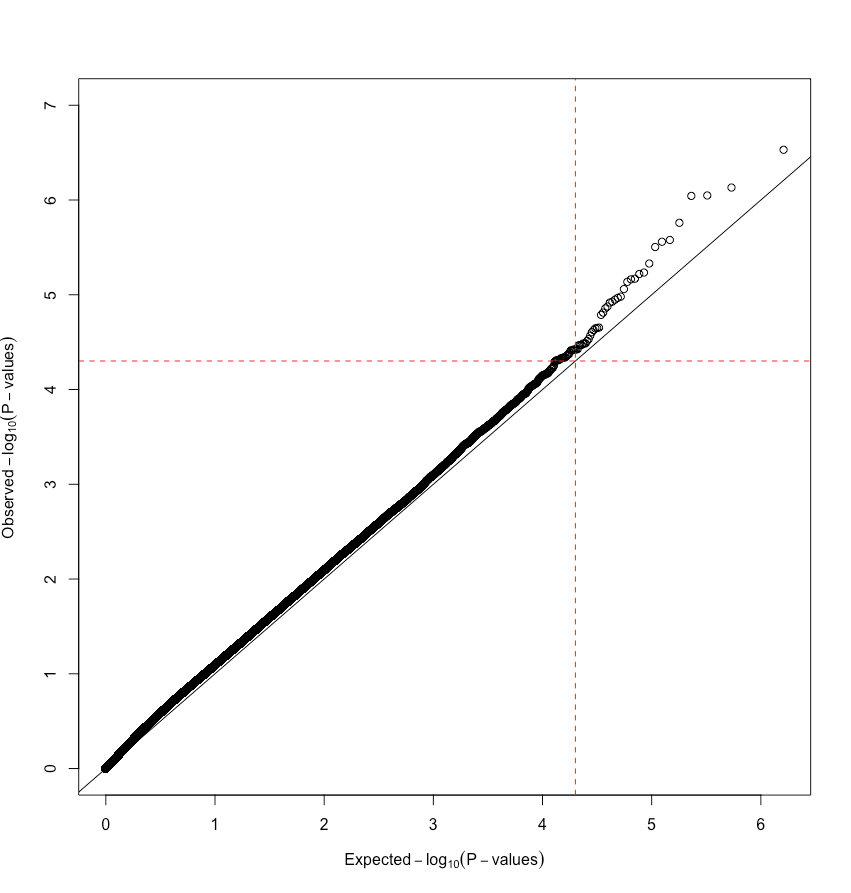

Supplement: Supplementary file 1 [file Image_1.TIFF]

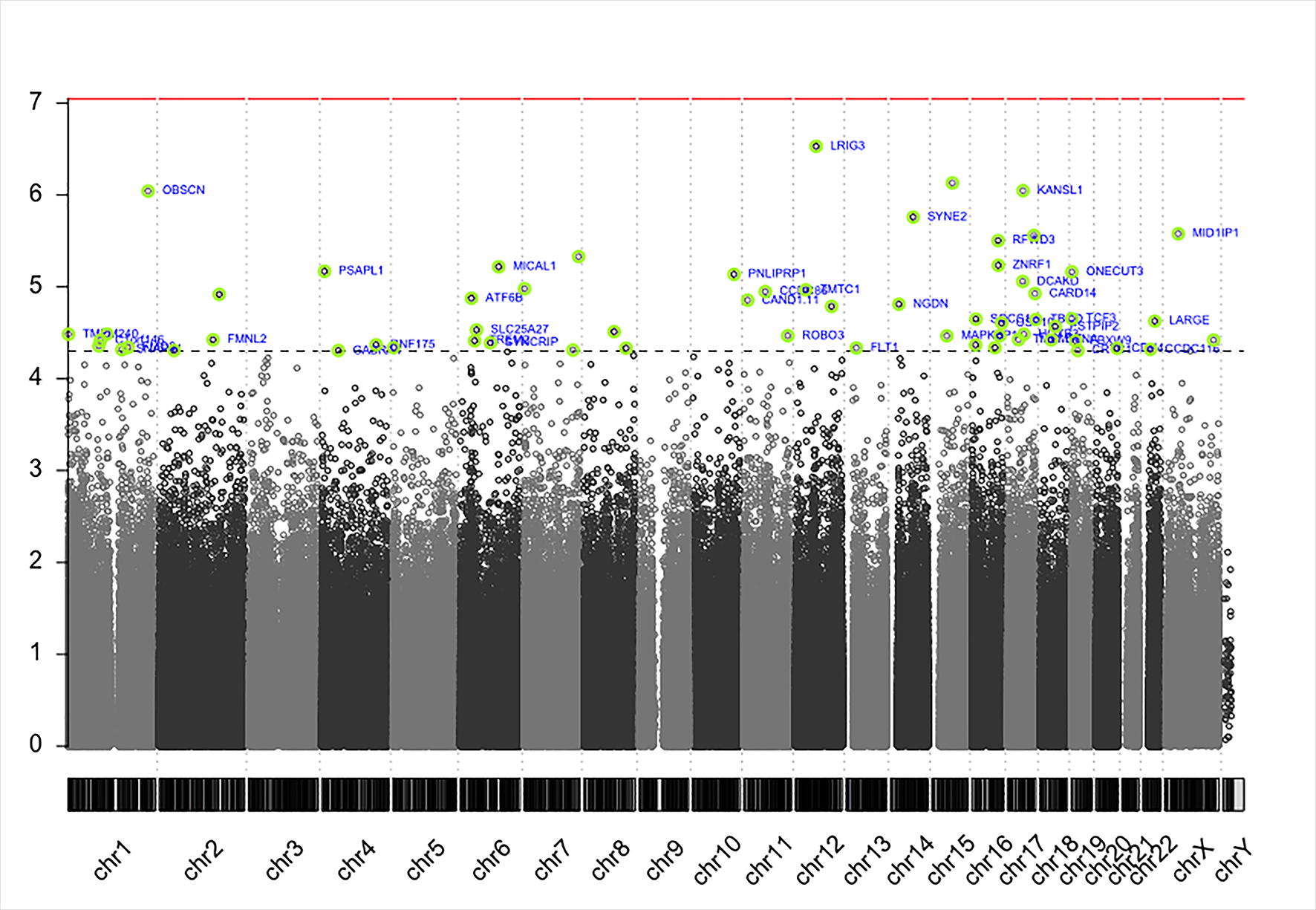

Supplement: Supplementary file 2 [file Image_2.TIFF]
